# Brain SPECT as a Biomarker of Neurodegeneration in Dementia in the Era of Molecular Imaging: Still a Valid Option?

**DOI:** 10.3389/fneur.2021.629442

**Published:** 2021-05-10

**Authors:** Rodolfo Ferrando, Andres Damian

**Affiliations:** ^1^Centro de Medicina Nuclear e Imagenología Molecular, Hospital de Clínicas, Universidad de la República (UdelaR), Montevideo, Uruguay; ^2^Centro Uruguayo de Imagenología Molecular (CUDIM), Montevideo, Uruguay

**Keywords:** SPECT, PET, biomarkers, Alzheimer's disease, dementia, neurodegeneration, low- and middle-income countries

## Abstract

Biomarkers are playing a progressively leading role in both clinical practice and scientific research in dementia. Although amyloid and tau biomarkers have gained ground in the clinical community in recent years, neurodegeneration biomarkers continue to play a key role due to their ability to identify different patterns of brain involvement that sign the transition between asymptomatic and symptomatic stages of the disease with high sensitivity and specificity. Both ^18^F-FDG positron emission tomography (PET) and perfusion single photon emission computed tomography (SPECT) have proved useful to reveal the functional alterations underlying various neurodegenerative diseases. Although the focus of nuclear neuroimaging has shifted to PET, the lower cost and wider availability of SPECT make it a still valid alternative for the study of patients with dementia. This review discusses the principles of both techniques, compares their diagnostic performance for the diagnosis of neurodegenerative diseases and highlights the role of SPECT to characterize patients from low- and middle-income countries, where special care of additional costs is particularly needed to meet the new recommendations for the diagnosis and characterization of patients with dementia.

## Introduction

Functional brain imaging includes a set of techniques that reveal biochemical, physiological, or electrical properties of the central nervous system. The most developed of these techniques are single photon emission tomography (SPECT), positron emission tomography (PET) and functional magnetic resonance imaging (fMRI). Of them, the first two are the most widely used and validated techniques in clinical practice, while the third is still limited to scientific research in dementia, and is the most appropriate modality for brain activation or connectivity studies. Magnetic resonance spectroscopy (MRS) is another functional technique that has clinical utility mostly in the evaluation of brain tumors, although it does not have yet defined clinical applications in dementia. SPECT and PET are nuclear medicine techniques that use radiopharmaceuticals for the evaluation of different functional phenomena (classically brain perfusion for SPECT or metabolism for PET), although today there is a plethora of tracers that allow the study of many molecular events in the brain.

SPECT is currently one of the most widely available imaging techniques for the study of brain function. It has been used successfully for the diagnosis of dementias since the 1980's, while PET made its way to the clinic in the following decade. Most recently, the evolution of nuclear techniques toward molecular imaging has allowed the *in vivo* detection of characteristic phenomena of neurodegenerative diseases such as disorders of dopaminergic function, beta-amyloid deposits or tau protein aggregates using specific tracers. Imaging of dopamine receptors, particularly dopamine transporter SPECT, already has consensual clinical applications in the study of encephalopathies associated with parkinsonism.

## Technical Considerations

### Brain SPECT

SPECT is a nuclear medicine imaging modality in which a gamma-emitter radiotracer is injected into the patient and tomographic images of its distribution are then obtained. The uptake of the radiotracer depends on the biochemical behavior of the tracer in the body (1).

The development of brain perfusion radiotracers consolidated the use of brain SPECT in the 1990's. The first radiotracers used were diffusible molecules (e.g., ^133^Xe), whose uptake depend on the arrival to the brain through the arterial system (cerebral perfusion) and on the concentration gradient between arterial blood and brain tissue. Through the application of kinetic analysis models, it was possible to obtain an absolut measure of the regional cerebral blood flow (rCBF) with ^133^Xe. Nevertheless, the low energy gamma rays emitted by ^133^Xe and its fast clearance from the brain determined a low spatial resolution. Few years later static tracers were developed (IMP, HIPDM, HMPAO, and ECD). These radiotracers are extracted by the brain on the initial arterial pass after peripheral i.v. administration and retained in proportion to the rCBF distribution. They are rather stable *in vivo* for at least 1 h, allowing images to be obtained for several minutes after injection. The most extensively used static radiotracers are ^99m^Tc-ECD (ethylcysteinate-dimer) and ^99m^Tc-HMPAO (hexamethyl propylene amine oxime) (2), with considerable technical, economic and logistical advantages (3). Their main characteristic is lipophilicity, which allows free diffusion through the blood-brain barrier with high extraction in the first pass through the cerebral circulation after the intravenous injection. This property determines an uptake that is proportional to the cerebral blood flow, maintaining a strong linear relationship at least up to 80 ml/min/100 g. After cellular uptake, these compounds are retained at the intracellular level for a long time (6 h for ECD and 4 h for the stabilized HMPAO kit) due to their transformation into hydrophilic compounds, with a fixed regional distribution that represents the functional state of the brain at the time of injection (2).

The close relationship between perfusion and neuronal metabolism is well-documented both in physiological conditions and in the vast majority of pathological processes, allowing the identification of hypometabolic regions through the corresponding decrease in perfusion using blood flow tracers (the same concept applies to fMRI). Tomographic images are acquired 45–60 min after injection for ECD and 60–90 min for HMPAO. Usually with a two-detector gamma camera a total acquisition time of 30 min is sufficient to achieve optimal image quality with a radiopharmaceutical dose of 925 MBq (25 mCi). The contrast of the images is usually higher with ECD than with HMPAO, and the dosimetry is slightly more favorable for ECD. The small differences in the normal brain distribution of both tracers are not considered relevant when interpreting clinical studies (2).

In addition to brain perfusion tracers, significant advances have been observed in recent years in the use of dopamine transporter (DAT) radiotracers. One of the most widely used DAT SPECT radiotracers is the tropane analog ^123^I-FP-CIT, which is used to demonstrate the presynaptic dopaminergic depletion in degenerative parkinsonisms. It has shown a good correlation with the severity and duration of Parkinson's disease, as well as clinical utility in the differential diagnosis between different forms of dementia. Given the low availability and high cost of ^123^I in low- and middle-income countries, many centers have opted for ^99m^Tc-labeled DAT radiotracers such as 99mTc-TRODAT, which has proven to perform well for the characterization of degenerative parkinsonisms (4–6). Typically, studies with ^99m^Tc-TRODAT require the administration of a dose of 925 MBq (25 mCi) and a delayed acquisition of 30 min at 3–4 h after injection to obtain images of adequate quality.

### Brain PET

PET is a nuclear imaging modality that allows obtaining tomographic images of the regional distribution in the brain of radiopharmaceuticals labeled with positron-emitting isotopes. The emitted positrons have a small trajectory in the body (usually a few millimeters) before each positron reacts with an electron of the subject in a process called annihilation, that results in the emission of two photons in opposite directions. PET scanners have rings of multiple detectors located around the patient that detect the coincidence of this pair of photons. By this process it is possible to infer the place where the positron was emitted and reconstruct tomographic images (7–9).

Positron-emitting radionuclides are produced in particle accelerators called cyclotrons and their half-life ranges from <2 to 110 min, which is acceptable for emission imaging. In the case of radiotracers labeled with ^18^F, which has a half-life of 110 min, the tracer can be produced and transported for injection and acquisition to distant centers. With ^11^C or ^15^O labeled radiotracers (20 min and 2 min half-life, respectively) the production and the study should be performed in the same center. The physical characteristics of this radioactive emission and its detection process provide PET with greater sensitivity and spatial resolution compared to SPECT. Nevertheless, it is worth to notice that this complex process and sophisticated equipment made the cost of PET several times higher than SPECT (1).

The most widely used radiopharmaceutical in clinical practice is the glucose analog ^18^F-fluorodeoxyglucose (^18^F-FDG). Brain metabolism depends particularly on glucose as the main energy substrate (10). This determines a high normal FDG uptake in the brain with high quality images, allowing a direct measurement of regional brain glucose metabolism. If dynamic image acquisition is performed, it is possible to quantify glucose consumption in absolute values using kinetic models. This technique is not used in clinical practice due to its complexity and the need for simultaneous arterial blood sampling. The typical acquisition starts 30–60 min after the injection of the tracer. The patient must remain in psychophysical rest for at least 30 min after the injection of the radiopharmaceutical due to the prolonged period of cerebral glucose uptake. The usual dose is 370 MBq (10 mCi) of ^18^F-FDG and image acquisition takes about 20 min (7).

It is also possible to measure absolute rCBF with different PET radiotracers, among which ^15^O-H_2_O stands out as the gold standard for non-invasive absolute quantification (11). The minimum requirement for this procedure is the acquisition of dynamic studies for their analysis using kinetic models. Since ^15^O has a 2-min half-life, it is possible to make several acquisitions on the same day. Typically, the dose to be administered is 300–500 MBq and the acquisition of each study does not take more than 10 min.

Since the early 2000's, amyloid imaging with PET started to gain ground as a new biomarker in patients with AD. The first radiotracer developed was the thioflavin T analog ^11^C-PIB (12) and later fluorinated analogs were incorporated (^18^F-florbetaben, ^18^F-florbetapir, and ^18^F-flutemetamol) extending the use of this modality in many countries. The imaging protocol and the quantification of the amyloid burden in the brain may vary depending on the radiotracer, but there are usually well-stablished criteria to determine if a patient has significative amyloid depositions. More recently, Tau tracers have been developed and included in the framework for research in AD (13).

## Clinical Utility of SPECT and PET in Alzheimer'S Disease

### Diagnosis of Alzheimer's Dementia

Although many patients with Alzheimer's disease (AD) have a characteristic clinical presentation, some forms of the disease may present with atypical symptoms. Diagnostic difficulties may rise in early disease stages, in atypical presentations or in clinical scenarios in which the differential diagnosis with other forms of dementia is challenging (14, 15). Murray et al. published a series of 889 cases of AD with histopathological confirmation, describing three well-defined subtypes with different clinical characteristics, of which the hippocampal-sparing subtype (11% of cases) was associated with atypical clinical presentation and previous diagnostic errors with higher frequency in comparison with the typical and predominantly limbic subtypes (16). Up to 25% of AD cases may show an atypical clinical presentation, supporting the use of functional neuroimaging biomarkers in diagnosis. Moreover, the hippocampal-sparing subtype represents a challenge for structural MRI, which usually relies on the identification of hippocampal atrophy as a hallmark for the diagnosis of AD (16).

SPECT and PET are the functional imaging modalities that have the most robust scientific support for their clinical application in dementias, and have been used successfully for several decades. The typical pattern of AD on SPECT or PET images is characterized by the presence of bilateral hypoperfusion or hypometabolism in the posterior parietal, temporal, and posterior cingulate cortex with preservation of the primary visual and sensorimotor cortex, basal ganglia, thalamus, brainstem and cerebellum. Generally, there is a lower degree of frontal involvement, which increases with the progression of the disease, usually sparing the motor cortex. The presence of this pattern has a high diagnostic accuracy, although other patterns with marked asymmetry, even unilateral, or with marked frontal involvement can be seen (2, 8, 17).

Usually there is a good correlation between SPECT/PET findings and symptom severity, although on certain clinical situations (including early-onset AD or in subjects with high intellectual level) this relationship may be more limited, with alterations that tend to be more severe in earlier clinical stages of the disease. This phenomenon is probably related to the concept of cognitive reserve. Those individuals with higher reserve are able to develop alternative strategies to compensate for the loss of functions related to the progress of the pathological process, thus delaying the onset of symptoms (18).

Studies on the diagnostic performance of SPECT in dementias showed variable results. The use of different radiopharmaceuticals, equipment, inclusion criteria and confirmation methods largely explain the variability of the findings. A systematic review by Dougall et al. evaluated the diagnostic accuracy of 45 studies performed with HMPAO in comparison with clinical diagnosis (DSM-III-R and NINCDS-ARDRA criteria) between the years 1988 and 2002 (19). The authors described a sensitivity of 77% and specificity of 89% for the diagnosis of AD compared to normal controls, 71 and 76% compared to vascular dementia, and 72 and 78% compared to frontotemporal dementia, using cross-sectional clinical diagnosis as the gold standard.

Regarding ^18^F-FDG PET, the meta-analysis of Patwardhan et al. included 15 articles published between 1989 and 2003, 10 of which included comparison with normal controls, showing sensitivity and specificity of 86% for the latter case (20). The final reference was clinical diagnosis in nine of the studies and histopathological confirmation in one. Five studies compared AD with other forms of dementia, showing similar sensitivity and significantly lower specificity (18–86%). Recently, Nestor et al. reviewed the evidence on the clinical use of ^18^F-FDG PET in diverse clinical scenarios, recommending the use of ^18^F-FDG for the differential diagnosis of other forms of dementia (21).

Of particular interest are the studies that evaluated the diagnostic accuracy of SPECT or PET using histopathological confirmation as a reference, since the assessment of the pathological hallmarks of AD is considered the most appropriate gold standard for research. Read et al. reported a sensitivity of 93% for SPECT compared with 73% for clinical diagnosis in a series of 27 patients that underwent autopsy (22). Bonte et al. published a series of articles using SPECT, in which the population studied progressively increased from 1993 and onwards. The final report of this series in 2011 included 73 patients and showed a sensitivity of 94%, specificity of 85%, positive predictive value of 92%, negative predictive value of 88%, and accuracy of 90% (23). Jobst et al. included 104 patients with dementia (80 of them with AD) and reported a sensitivity, specificity, and accuracy of 89, 80, and 83%, respectively (24).

It is important to notice that some of these studies were published more than 20 years ago and used the equipment available at that time, with a performance likely below the current standards in the field. Since then, many technical advances have been incorporated into clinical routine, including iterative reconstruction methods (OSEM), scatter correction, attenuation correction using CT maps in hybrid equipment and resolution recovery. All of them have contributed to improving the quality of the images with a probable positive impact on the diagnostic performance of SPECT. El Fakhri et al. demonstrated the advantages of several of these improvements in patients with mild cognitive impairment (MCI) (25).

Regarding PET, Silverman et al. published results of a multicenter study that included 138 patients (97 patients with AD, 23 with non-AD neurodegeneration and 18 with no neurodegenerative dementia), one of the largest series existing to date, and reported values of 94% sensitivity, 73% specificity, and 88% accuracy (26). Previously, Hoffman et al. published an institutional series of 22 patients with AD and other dementias in which they described a sensitivity of 88% and a specificity of 67% (27). Jagust et al. in a series of 44 patients (including 20 patients with AD, 9 normal controls, and patients with mixed dementia and LBD among others) reported values of 84 and 74%, respectively (28).

### Additional Value of SPECT and PET in the Clinical Context

Most of the studies previously mentioned included SPECT or PET interpretation blinded to clinical information, which does not represent the usual situation in practice, in which clinical information is used to support image interpretation, improving the diagnostic performance. The data might therefore be interpreted as a conservative estimate of the usefulness of these modalities as an isolated diagnostic tool, and probably do not reflect their true impact as complementary tools to clinical evaluation. The importance of a diagnostic test often lies in the additional information that provides over the already available clinical data. Thus, even assuming that the diagnostic performance of SPECT/PET in AD could be similar to neuropsychological tests, the combination of both can increase the diagnostic accuracy. In this regard, Jagust et al. (29) analyzed data from the aforementioned work by Jobst et al. (24) evaluating the additional impact of SPECT on clinical diagnosis and reported that a positive SPECT increased the probability of AD in histopathology from 84 to 92% for the diagnosis of probable AD and from 67 to 84% for the diagnosis of possible AD. A clinical diagnosis of possible AD with a positive SPECT was associated with the same probability of AD as the clinical diagnosis of probable AD, whereas a diagnosis of probable AD with positive SPECT implies a very high probability of AD on neuropathology. Claus et al. in a study carried out in a community population found that when the previous probability of AD was 50%, the additional information provided by SPECT increased the diagnostic certainty by 34% (30). Silverman et al. in a retrospective analysis of 167 patients studied with FDG PET with an average clinical follow-up of 3 years reported that when the initial clinical evaluation predicted progressive deterioration (based on suspected neurodegenerative disease), 94% of the patients with a positive PET finally deteriorated while the percentage dropped to 25% when PET was negative (31). According to Jagust et al., the clinical diagnosis was associated with a 70% probability of AD in histopathology, while this value increased to 84% with a positive PET and decreased to 31% with a negative PET. A clinical diagnosis not compatible with AD was associated with pathology of AD in 35% of the patients, which increased to 70% with a positive PET (28).

### The Value of Quantification

Many studies have evaluated the impact of semi-quantitative methods on the diagnostic performance of SPECT in dementia, demonstrating an increase in accuracy with respect to visual interpretation and a decrease in interobserver variability. Voxel-based analysis transforms the images of each subject to a common stereotaxic space and then applies statistical tests to identify groups of voxels that differ between groups of patients or between patients and normal controls. This analysis can be applied similarly to SPECT and PET images. Originally developed for the use in research studies, it has gained acceptance in clinical practice to determine the statistical deviation of images of an individual subject from a database of normal controls. The two most widely used tools are Statistical Parametric Mapping (SPM) and Tridimensional Stereotactic Surface Projections (3D-SSP), both of which are freely accessible. It has been shown that with the use of these tools the sensitivity and specificity of SPECT can be increased to above 90 and 85%, respectively for the diagnosis of AD, even in early stages (32, 33). Alternatively, a region of interest (ROI) approach can be used to compare the uptake in predefined regions of the brain with normal controls. The defined ROIs should include the brain areas most frequently involved in the different types of dementia (such as posterior parietal cortex, precuneus and posterior cingulate in AD, the dorsolateral, medial and ventral frontal cortex and anterior cingulate in frontotemporal dementia, or the posterior parietal and occipital cortex in Lewy body disease) Different reference regions can be used for intensity normalization when applying a semi-quantitative approach. Usually, a region not affected in the disease and with low variability between patients and controls is chosen (for instance the pons, cerebellum or the whole brain for AD).

The semi-quantitative approach is widely available and usually easy to implement, even in centers from low- and middle-income countries. In contrast, the full quantitative approach requires the acquisition of dynamic studies, the implementation of kinetic analysis, a specific training of the staff and, in some cases, arterial blood sampling. All these issues have led to a more widespread use of the semi-quantitative approach in clinical practice, while full quantification is usually reserved for research purposes.

### Prognostic Value for Conversion From Mild Cognitive Impairment to Alzheimer's Dementia

Of particular importance is the potential of functional imaging techniques for the diagnosis of AD in the early disease stages, such as MCI. The identification of subjects at high risk of evolution to AD at the MCI stage can allow the intervention in the initial clinical phase of the disease with the aim of delaying the evolution of symptoms and the appearance of dementia. The presence of hypoperfusion in SPECT or hypometabolism in PET in the posterior parietal cortex, precuneus, and posterior cingulate in patients with MCI has been consistently associated with an increased risk of progression to AD (34–36). In particular, posterior cingulate has been identified as a region that provides high discriminative power. SPECT reports showed an accuracy of ~75–80% when using image quantification techniques (37–39). Nobili et al. described a sensitivity of 81% and specificity of 86% for hippocampal hypoperfusion as a predictive marker of conversion to AD. Silverman et al. reported maintained sensitivity and specificity (95 and 71%, respectively) for PET in the subgroup of patients with mild AD in their retrospective multicenter study of patients evaluated for dementia with histopathological confirmation (26). A meta-analysis by Yuan et al., which included 24 studies with a total of 1,112 patients, found sensitivity and specificity values of 89 and 85%, respectively for PET, 84 and 70% for SPECT, and 73 and 81% for MRI, for prediction of conversion from MCI to AD (40). A review by Frisoni et al. reported pooled sensitivity and specificity for conversion of MCI to AD of 76 and 74%, respectively for PET and 78 and 64%, respectively for SPECT (41).

## Clinical Use of SPECT and PET in the Differential Diagnosis of Dementia

### Frontotemporal Dementia

The presence of anterior temporal and frontal hypoperfusion or hypometabolism typically identifies patients with frontotemporal dementia (FTD), as opposed to the posterior temporoparietal pattern characteristic of AD. Using this criterion, a systematic review by Yeo et al., which included 10 studies that used SPECT, reported sensitivity and specificity of 80% using clinical follow-up as a reference (42). Even though there may be varying degrees of posterior cortical involvement in FTD, as well as atypical AD presentations with marked frontal involvement, in general, the balance between the severity of the anterior and posterior cortical involvement provides good results to discriminate both clinical entities. Sjögren et al. reported a sensitivity of 88% and a specificity of 79% for HMPAO SPECT using an anterior-to-posterior ratio quantification strategy to differentiate FTD from other forms of dementia. Specificity increased to 96% compared to early-onset AD (43). The relative preservation of the posterior cingulate cortex in FTD and the indemnity of the primary sensorimotor cortex and subcortical gray structures in AD, have been described as other useful findings. Méndez et al. retrospectively evaluated 134 patients referred for clinical suspicion of FTD and reported sensitivity and specificity of 37 and 100% for clinical follow-up at 2 years, 64 and 70% for MRI and 91 and 75% for SPECT/PET (44). Read et al. studied 27 patients with dementia (including eight patients with FTD and 11 with possible or probable AD) and neuropathological confirmation and found that SPECT was able to correctly classify 93% of the cases, while clinical evaluation was successful in classifying the patients in 74% of the cases (22).

SPECT and PET can identify dysfunctional patterns that contribute not only to the differential diagnosis between AD and FTD, but also to the diagnosis of the different FTD variants. The behavioral variant of FTD presents with a predominant medial prefrontal, anterior cingulate, middle and inferior frontal gyri and superior temporal hypometabolism (45). The semantic variant of primary progressive aphasia presents with anterior temporal hypoperfusion or hypometabolism with a clear left predominance, while the non-fluent variant shows involvement of the left frontal operculum. The logopenic variant is characterized by defects of the left posterior temporoparietal cortex and the underlying pathology is more likely AD.

### Lewy Body Dementia

**Figure 1:**
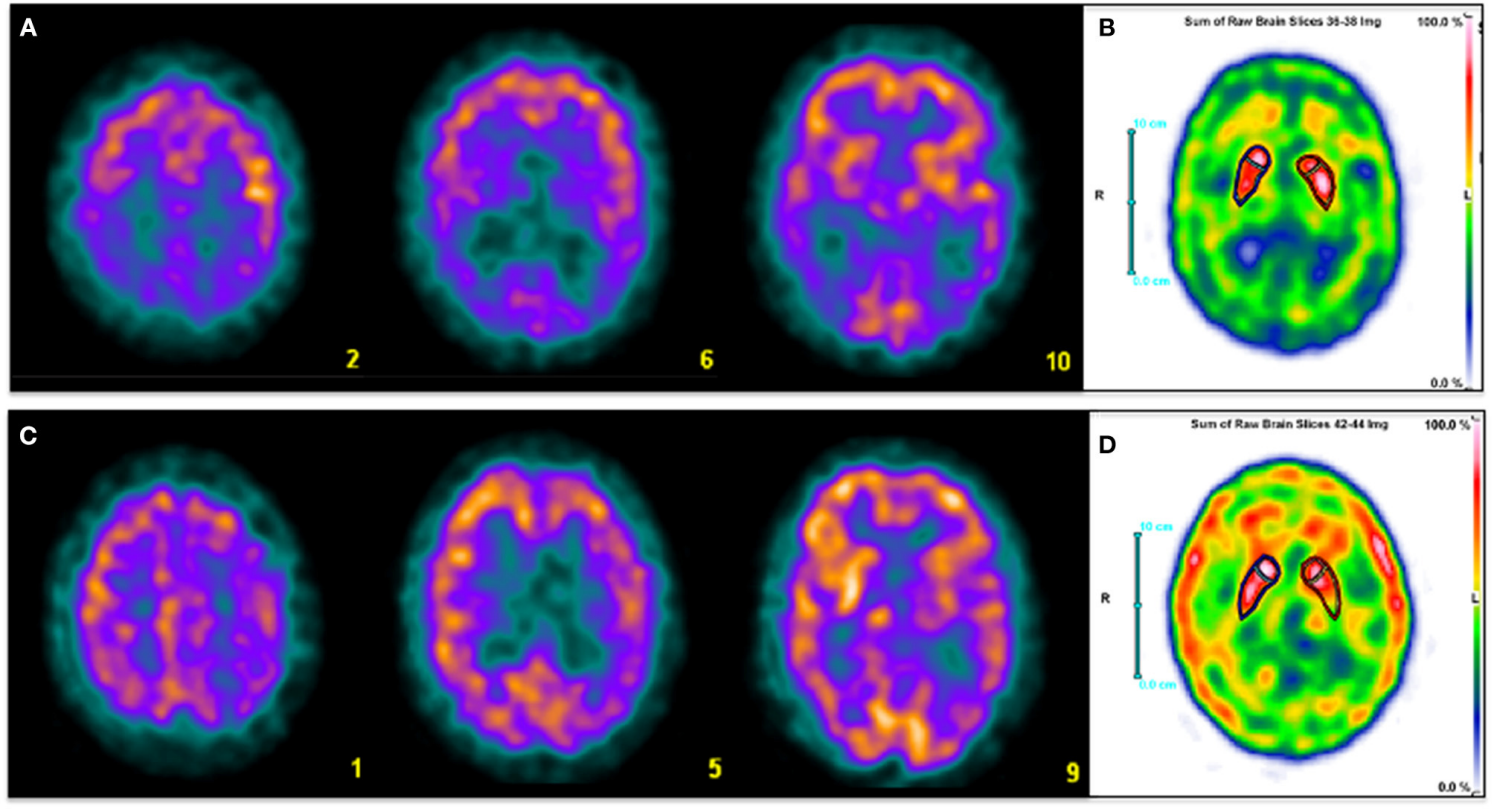
Three selected transaxial slices of the perfusion SPECT **(A,C)** and a sum of three consecutive axial slices at the level of basal ganglia of DAT SPECT **(C,D)** of two patients referred for clinical evaluation of cognitive impairment. Superior row corresponds to a 59-year-old man with fluctuating cognitive impairment with frontal-subcortical profile in the neuropsychological examination. A typical pattern of LBD is depicted, with severe bilateral posterior parietotemporal and occipital hypoperfusion **(A)**. DAT imaging shows mildly reduced presynaptic dopamine transmission in right putamen and left caudate nucleus **(B)**. Inferior row shows a 63 year-old man with amnestic cognitive impairment and right extra-pyramidal signs. Diffuse hypoperfusion involving left parietal, frontal and temporal cortex, ipsilateral basal ganglia and thalamus **(C)** is associated with reduced left striatal dopamine transmission with posterior putamen predominance **(D)**. Findings are consistent with CBD. Clinical diagnosis was made based on perfusion SPECT in both patients. DAT SPECT images were performed later for academic purposes.

Lewy body dementia (LBD) shows a pattern of hypoperfusion or hypometabolism that usually involves the posterior parietotemporal cortex (similar to AD) but sparing the posterior cingulate and usually with extension to the occipital cortex. Occipital involvement, particularly of the primary visual cortex, has been identified as the most valuable sign for differentiating LBD from AD. Lobotesis et al. reported sensitivity of 65% and specificity of 87% for the differential diagnosis between LBD and AD by SPECT using this criterion (46). Shimizu et al. reported a sensitivity and specificity of 85% using voxel-based analysis (47). PET studies showed similar, and in some cases superior, diagnostic performance. Minoshima et al. reported a sensitivity of 90% and specificity of 80% for hypometabolism of the occipital cortex in the differential diagnosis with AD (48). Based on the involvement of the occipital cortex and the relative preservation of the posterior cingulate (posterior cingulate island sign), the diagnostic criteria of McKieth et al. (49, 50) recognize the role of SPECT/PET as a supportive marker for the diagnosis of LBD. It has to be considered that occipital involvement may be present in other pathologies such as Parkinson's dementia and may be absent in a non-negligible percentage of patients with LBD who show a perfusion or metabolic pattern very similar to AD. LBD is characterized by striatal dopaminergic deficiency that is related to parkinsonism, one of the core symptoms of the disease. The decrease in DAT density at the presynaptic dopaminergic terminal is a consequence of the degeneration of nigrostriatal neurons, a phenomenon that can be measured through specific SPECT tracers. The most widely used of them is ^123^I-FP-CIT (ioflupane). This image modality allows the differential diagnosis with AD with high performance. The reduction in striatal uptake of ^123^I-FP-CIT was able to distinguish LBD and AD with a sensitivity of 88% and specificity of 100% in a series of 20 patients with histopathological confirmation, while the initial clinical diagnosis showed a sensitivity of 77% and specificity of 42% (51). In a multicenter study that included 326 patients using clinical diagnosis as a reference, McKieth et al. reported sensitivity of 78% and specificity of 90% (52). The 2017 consensus criteria for LBD consider reduced striatal uptake in DAT SPECT/PET as an indicative biomarker of the disease (50). DAT SPECT has not shown the same utility for the differential diagnosis between LBD and FTD, since in the latter the presence of extrapyramidal signs is not uncommon. In this regard, perfusion SPECT has much greater utility. Although ^123^I-FP-CIT may not be available in many low- and middle-income countries due to its high cost and the low availability of ^123^I, other alternatives like ^99m^Tc-TRODAT-1 have shown excellent correlation with ^123^I-FP-CIT in various diseases associated with parkinsonism. Although DAT imaging has demonstrated greater accuracy than perfusion/metabolic imaging in the differential diagnosis between LBD and AD, this particular scenario is not always present in clinical practice. The differential diagnosis may often present with different types of dementia like FTD, parkinsonism-related dementias, vascular dementia, and others, and DAT imaging is less likely to solve the problem than perfusion/metabolic imaging in these cases. Moreover, it is not clear if the impact of DAT imaging in LBD is the same when clear features of parkinsonism are present or not. It seems reasonable to hypothesize that reduced uptake of a DAT tracer in the context of parkinsonian signs is less likely to provide relevant additional information, and perfusion/metabolic imaging can make a more considerable difference. Taking these considerations into account, it may be reasonable to start with perfusion/metabolic imaging in many cases, and reserve DAT imaging for a second stage when necessary, enabling significant savings due to the higher cost of the latter. This is exemplified in Figures 1A,B.

### Parkinsonism-Related Dementias

At a certain point of the disease, up to 30–40% of patients with Parkinson's disease (PD) may have dementia symptoms and up to 75–80% of patients with PD may develop dementia during a 10 year period (15, 53, 54). The cognitive impairment that usually presents in PD is typically of the frontal-subcortical type and do not reach the MCI stage, while those cases that progress to dementia are characterized by predominant posterior cortical symptoms (15). This phenomenon can be identified by SPECT or PET by the presence of a dysfunctional pattern similar to that of AD. Although more frequent extension to the occipital cortex and greater preservation of the medial temporal structures have been described in PD dementia in comparison with AD, the findings in both diseases may be indistinguishable (55). The pattern of PD dementia can be even more difficult to distinguish from AD than the one of LBD. However, these diseases are usually differentiated by the temporal course of the appearance of dementia symptoms and extrapyramidal signs (50). Although the vast majority of parkinsonisms are PD, the so-called atypical parkinsonisms can represent up to 15% of the cases and their clinical diagnosis can be particularly complex. Among them are multisystemic atrophy (MSA), progressive supranuclear palsy (PSP) and corticobasal degeneration (CBD) (55, 56). These diseases are associated with a higher prevalence of dementia and are characterized by a frequent compromise of the post-synaptic dopaminergic system, unlike PD. Post-synaptic D2 receptor SPECT may be useful for the differential diagnosis between PD and atypical parkinsonism, although some reports indicate that its diagnostic accuracy would be suboptimal (57, 58). Furthermore, its high cost, low availability and its inability to differentiate between different forms of atypical parkinsonism considerably limit its use in clinical practice. Various publications have shown that perfusion SPECT or FDG PET (associated with DAT imaging) have a higher performance for the differential diagnosis between these diseases (57, 59). Atypical parkinsonisms are characterized by the presence of striatal hypoperfusion or hypometabolism, unlike PD. On the other hand, cerebellar involvement orients to MSA, while a frankly asymmetric cortical-subcortical pattern is characteristic of CBD (Figures 1C,D), and PSP shows alterations in the superior and medial frontal cortex, thalamus and pons (55, 60). Thus, perfusion/metabolic imaging can not only confirm the diagnosis of atypical parkinsonism revealing striatal involvement but also contribute to the differential diagnosis of the specific disease.

### Vascular Dementia

In the diagnosis of vascular dementia (VD), structural images (especially MRI) play a leading role (15, 61). SPECT and PET have traditionally been reserved for equivocal cases. However, 15–20% of patients with VD have mixed dementia, more frequently VD and AD. In these cases, functional images are useful to confirm or rule out the presence of associated AD. Multi-infarct dementia is characterized by multiple asymmetric focal defects with well-defined borders scattered in the cerebral cortex, frequently in borderline territories of the cerebral arteries, which can be associated with subcortical and cerebellar defects. Dementia due to strategic infarct shows a well-defined extensive defect in a specific vascular territory. The presence of crossed cerebellar diaschisis is frequent in both cases and is considered an element of diagnostic value. When the strategic infarct is located in subcortical structures such as the internal capsule or the thalamus, cortical hypoperfusion is usually associated due to disruption of the thalamocortical projections (diaschisis). Subcortical vascular dementia is characterized by hypoperfusion of subcortical gray structures associated with diffuse moderate cortical involvement that mostly affect the frontal cortex, as a result of diaschisis phenomena associated with white matter abnormalities. The presence of bilateral posterior temporoparietal perfusion defects, characteristic of AD, differs from the typical patterns of VD, allowing the differential diagnosis between both diseases as well as the diagnosis of association between both. Some authors like Nagata et al. state that the information provided by SPECT/PET is useful in the differential diagnosis between VD, AD and mixed dementia and should be taken into account in the diagnostic guidelines (61). SPECT with vasodilator stimulation with acetazolamide has shown to be capable of providing additional information in the differential diagnosis of VD with AD, increasing the performance of images at rest (62).

Finally, SPECT and PET also have diagnostic utility in other less frequent dementias, including traumatic brain injury, AIDS dementia, autoimmune or paraneoplastic encephalitis, neurolupus, Behcet's disease, exposure to neurotoxins, psychiatric diseases, Creutzfeld-Jacob disease, Huntington's disease and other low incidence degenerative encephalopathies (2, 8, 11, 63).

A separate chapter deserves late-life depression (which occurs frequently with a predominance of cognitive symptoms), in which the identification of involvement of the posterior temporoparietal cortex indicates a high probability of underlying AD.

## Comparison of SPECT and PET With Other Biomarkers of Neurodegeneration

Structural imaging is routinely used in the evaluation of patients with dementia. CT or MRI have been proven to change clinical diagnosis in 19% of patients and clinical management in 15% of them, even with conservative use, usually by detecting stroke, tumors or other diseases not suspected as the cause of symptoms (64). However, visual interpretation of structural imaging does not reliably detect neurodegenerative processes in early stages (65). Hippocampal volume measure is one of the best-established markers for AD. Pucci et al. reported 79% sensitivity and 69% specificity in distinguishing AD patients from normal controls, although the mean MMSE score was 15 in this population (66). Jack et al. found sensitivity of 82% and specificity of 80%, with similar values in a subset of milder patients (67).

The studies comparing the performance of different biomarkers are more limited. In a meta-analysis by Yuan et al., FDG PET performed slightly better than SPECT and MRI in the prediction of conversion from MCI to AD, with similar results for SPECT and MRI (40). Frisoni et al. found lower sensitivity and specificity for MRI compared to PET and SPECT (41). These results are probably related to the low specificity of medial temporal atrophy, which occurs in a proportion of cognitively healthy older people, as well as other pathological conditions (68). The authors also found that diagnostic accuracy of imaging biomarkers is highly dependent on how the biomarker is measured, and they identified four different metrics for medial temporal lobe atrophy on MRI. Standard operating procedures are needed to obtain reliable results in clinical practice and are not always available, particularly in developing countries (69). It should be noted that the accuracy of hippocampal measure is dependent upon, and influenced by, the dementia state and disease severity of patients studied. A mildly affected brain would be much more difficult to diagnose than a severely demented one. Moreover, quantification of hippocampal volumes is time-consuming and requires considerable neuroanatomic expertise or specific software. Only a few MRI centers in our country perform this procedure.

The analysis of CSF for increased concentrations of tau proteins is another recognized biomarker for AD. Markers of tau accumulation include increased total tau or phosphorylated-tau (p-tau) and are clearly associated with AD pathology. While changes in tau can also reflect general damage to neurons and synapses, p-tau occurs solely in AD and is therefore a more specific biomarker. Together with low CSF Ab42, elevated CSF tau provides a high likelihood of progression to AD in patients with MCI. A large meta-analysis by Mitchell that included 19 studies with a total of 2.300 AD patients and normal controls reported sensitivity of 78%, specificity of 88%, positive predictive value of 93% and negative predictive value of 73% (70). The same meta-analysis also included 18 studies with AD and non-AD dementia patients and found values of 72, 78, 86, and 58%, respectively for distinguishing both groups.

It is important to emphasize that standardization of CSF biomarkers is still limited and results often vary between different laboratories (71). Each laboratory must define its own normal limits and, ultimately, it will be necessary to define well-established normative values, which is still in process (72). The need to perform a lumbar puncture, a procedure that is regarded as complicated, time-consuming and invasive for many clinicians, is another well-recognized limitation of these biomarkers. Recent developments enabled the measurement of AD biomarkers in blood samples. Plasma p-tau has shown analytical validity and first evidence of clinical validity. While the results are very promising, sufficient data about the effect of covariates on the biomarker measurement, assay comparison and cut-off criteria are still lacking (73).

Regarding comparison with other biomarkers, a review and meta-analysis by Bloudek et al., finally including 119 studies, found that FDG PET was most accurate than SPECT and p-tau in discriminating AD from normal controls with sensitivity of 90% and specificity of 89%. Compared to demented controls (including MCI), PET sensitivity was maintained at 92% and specificity decreased to 78%. For discrimination of AD from non-AD dementias (excluding MCI), p-tau and SPECT had nearly identical performance with sensitivity of 79% and specificities of 80 and 81%, respectively (74).

A recent systematic review by Fink et al. found that individual CSF biomarkers and biomarker ratios had moderate sensitivity (62–83%) and specificity (53–69%) for distinguishing neuropathologically defined AD from non-AD pathology, while β-amyloid 42 (Aβ42)/p-tau ratio, total tau (t-tau)/Aβ42 ratio, and p-tau appeared more accurate than Ab42 and t-tau alone. Median sensitivity and specificity for amyloid PET were 91 and 92%, respectively, 89 and 74% for FDG PET, 64 and 83% for SPECT, and 91 and 89% for medial temporal lobe atrophy on MRI. Sensitivity and specificity of MRI was considerably lower for distinguishing AD from other specific types of dementia like LBD or FTD (75).

In one of the few studies that compared multiple biomarkers in the same group of patients, Morinaga et al. included 207 patients with probable AD from a single memory clinic. AD findings were observed in 77.4% of all AD patients with MRI, 81.6% with SPECT, 93.1% with FDG PET and 94.0% with CSF biomarkers. At the stage of Clinical Dementia Rating (CDR) 0.5, sensitivity was 90.0% for CSF, 80.8% for SPECT, 71.4 for FDG PET and 65.5% for MRI. At the stage of CDR 1, FDG PET (96.7%) and CSF biomarkers (95.5%) were the most sensitive. At CDR 2, all biomarkers showed high sensitivity (76).

Although MRI and CSF biomarkers have shown similar performance to SPECT and FDG-PET in distinguishing AD from non-AD patients, they cannnot reliably differentiate between different types of non-AD dementia, reducing their applicability in clinical practice with respect to functional imaging techniques. The availability of these biomarkers in developing countries is limited for several reasons already mentioned. While PET suffers from similar limitations because of its high cost, brain SPECT is available in all nuclear medicine facilities at a much lower cost.

## Direct Comparison Between SPECT and PET in Alzheimer's Disease

The technical characteristics of PET determines an overall higher performance over SPECT, in particular its greater sensitivity (referred to the detection efficiency of the radioactive emission) and spatial resolution. Even though significant, the differences in spatial resolution between both modalities are not of great magnitude, with values of 3–4 mm for PET and 5–8 mm for SPECT. The spatial resolution of PET is limited to 1–2 mm due to the positron emission range, while SPECT has no theoretical limitations in this regard, which has led to sub-millimeter resolutions in small animal equipment, exceeding the spatial resolution of their PET analogs. Dedicated brain SPECT cameras have the same spatial resolution as PET, although their availability is very limited and there has been no significant expansion of its use in clinical practice.

A critical question is to what extent the technical differences between both modalities influence the clinical diagnosis of neurodegenerative diseases, that is, whether or not they translate into considerable differences in diagnostic performance. The answer requires a critical review of the available literature. Considering systematic reviews or meta-analyses of the diagnostic value of both techniques in AD, Dougall et al. reported a sensitivity of 77% and specificity of 89% for SPECT (19) while Patwardhan et al. including studies from the same time period reported a 86% sensitivity and specificity for PET (20). These results indicate a higher sensitivity and slightly lower specificity for PET.

Frisoni et al. reviewed the diagnostic and prognostic accuracy of different AD imaging biomarkers (amyloid PET, FDG PET, perfusion SPECT and MRI) and their operating procedures or metrics (visual analysis and different quantitative and semiquantitive approaches) (41). Interestingly, they found that different metrics can account for equal or more variation in the accuracy than the types of markers used. Pooled sensitivity and specificity for AD diagnosis for all metrics were 86 and 84%, respectively for PET and 76 and 84%, respectively for SPECT. Quantification increased SPECT sensitivity more than 10% to the same range as PET while it had very little effect on PET sensitivity (41).

Clinical diagnosis was used as a reference in most of the publications included in review studies. Very few studies included neuropathological confirmation as a reference, considered the most appropriate gold standard. The studies by Jobst et al. (24) and Bonte et al. (23) show pooled sensitivity, specificity, and accuracy of 91, 81, and 85% for SPECT. According to the studies of Silverman et al. (26, 77) and Hoffman et al. (27), pooled values of 92, 70, and 85% (sensitivity, specificity, and accuracy, respectively) are obtained for PET (the study by Jagust et al. which reported lower values for PET in 2007 is not included). According to these data, compared to neuropathological confirmation, the accuracy is similar for both techniques.

There are also few studies that have directly compared SPECT and PET in the same sample of patients. Kuwabara et al. studied nine patients with AD with different nuclear imaging modalities including ^123^I-IMP and HMPAO SPECT, and ^15^O-H_2_O and ^18^F-FDG PET. They described that even though there was a slightly lower performance of SPECT for the detection of areas with mild hypoperfusion, both SPECT and PET were able to detect parietal abnormalities in all AD patients (78). Messa et al. studied 21 patients with probable AD by both techniques and found bilateral posterior temporoparietal involvement in 90% of cases with SPECT and 100% with PET (79). Mielke et al. in the same year, on a similar sample of patients, reported that PET discriminated AD patients from normal controls only marginally better than SPECT (80). Herholz et al. studied 26 patients with mild to moderate AD and six normal controls with SPECT and PET and analyzed the results using voxel-based analysis (SPM), demonstrating a correlation coefficient of 0.90 for posterior temporoparietal cortex and posterior cingulate defects in both studies (81). The defects were more pronounced on PET images, but both techniques were able to adequately separate all patients from normal controls. Scatter correction was not used for SPECT studies at that time, and it is known that this procedure, widely available today, allows to considerably increase the contrast of the images. Döbert et al. studied 24 patients with clinical suspicion of dementia onset (12 of them with MCI) using clinical follow-up as a reference and found greater sensitivity for PET without differences in specificity, although the sensitivity values were low for both techniques (82). Nihashi et al. found no significant differences between FDG PET and IMP SPECT in 14 patients with moderate probable AD relative to normal controls using statistical analysis with 3D-SSP (83). More recently, Ito et al. published a comparative series using FDG PET and ^99m^Tc-ECD SPECT in 55 patients with cognitive impairment classified as probable AD (*n* = 28), MCI due to AD (*n* = 12) or no AD (*n* = 15) according to the NIA-AA criteria recommended for research studies, including MRI and ^11^C-PIB amyloid imaging (84). The results were interpreted by three independent observers with variable experience. The image acquisition and processing techniques used reflect the state of the art of SPECT and PET today. The diagnostic accuracy was in the range of 60–70% for both techniques and was practically identical, with no significant differences between them. The low performance demonstrated by both techniques in this study with respect to previous reports may be related to the fact that all the patients had cognitive impairment, and normal controls were not included in the analysis. In the same year, O'Brien et al. compared 38 patients with AD, 30 with LBD and 30 normal controls (85). Specificity for dementia/no-dementia was 85 and 90%, respectively for PET and 71 and 70%, respectively for SPECT. The patients in this study were not clinically referred, limiting the applicability of the results, and the authors relied on clinical diagnosis of probable AD or LBD without follow-up. Moreover, the inclusion of patients with LBD, that can be difficult to distinguish from AD (particularly on SPECT images because of the high normal uptake of the occipital cortex), may have influenced the results. Ferreira et al. studied 20 patients with mild AD and 18 normal controls, showing a similar accuracy for SPECT and PET (68–74 and 68–71%, respectively), highlighting the key role of SPECT in the study of patients with AD (86). In a larger study recently published, Nadebaum et al. evaluated the diagnostic performance of SPECT and PET in 126 patients with MCI and dementia, including amyloid PET information as a reference for the final diagnosis (87). They showed higher sensitivity for PET (75.8 vs. 42.9 for SPECT) but lower specificity (74.3 vs. 82.9% for SPECT). It is important to notice the very low sensitivity reported for SPECT in this study, much lower than expected based on the previous literature. Part of the explanation could be due to the presence of MCI in more than half of the patients included, that usually have more subtle functional alterations more easily recognizable in PET images.

**Figure 2:**
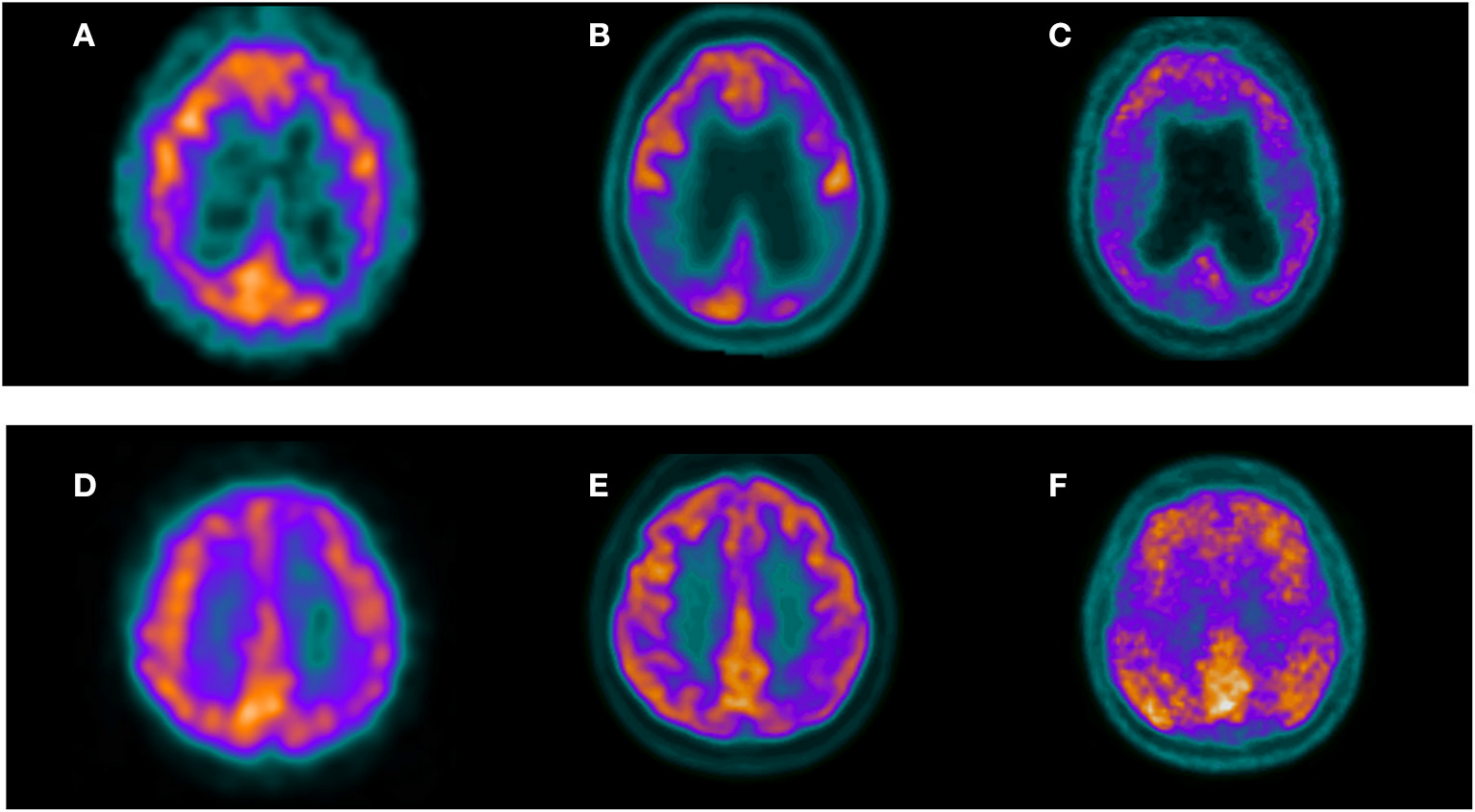
Three selected transaxial slices of the perfusion SPECT, ^18^F-FDG PET, and ^11^C-PIB PET of two patients referred for clinical evaluation of cognitive impairment. Superior row corresponds to a 63-year-old female with a mild cognitive impairment. MMSE was 27. Both perfusion SPECT **(A)** and ^18^F-FDG PET **(B)** showed hypoperfusion/hypometabolism in the bilateral parietal cortex with a typical pattern of AD. Amyloid PET in this patient **(C)** showed significant cortical amyloid deposits. Inferior row shows a 55-year-old female referred for evaluation of probable AD. Both perfusion SPECT **(D)** and ^18^F-FDG PET **(E)** showed a left posterior parietal hypoperfusion/hypometabolism suggestive of AD. ^11^C-PIB PET in this patient **(F)** confirmed cortical amyloid deposits.

All this data collectively demonstrates that even though there can be a slight advantage in favor of PET in terms of sensitivity, the diagnostic performance of SPECT and PET in AD is fairly similar. Figure 2 shows the example of two patients clinically referred in which both SPECT and PET allow reaching the diagnosis. It is possible that the differences are more evident in patients with MCI, which can show less perfusion/metabolic abnormalities in comparison with patients with dementia. In patients with MCI a difference in accuracy of about 10% in favor of PET in comparison with SPECT has been described, although more information is needed to finally conclude about the difference in diagnostic performance of both modalities in this clinical scenario. Table 1 shows a summary of the previously mentioned articles directly comparing SPECT and PET in the same group of patients.

**Table 1:** Summary of studies directly comparing SPECT and PET performance in the same group of patients.

**References**	**Subjects**	**Number of patients**	**PET sensitivity/** **specificity (accuracy)**	**SPECT sensitivity/** **specificity (accuracy)**	**Comments**
Kuwabara et al. (78)	AD, FTD and VD	9 AD, 3 FTD and 5 VD	–	–	Both SPECT and PET identified parietal abnormalities in all AD patients
Messa et al. (79)	Probable AD and normal controls	21 AD, 20 NC	100/-	90/-	
Mielke et al. (80)	Probable AD, vascular dementia and normal controls	20 AD, 12 VD, 13 NC	80/100	80/65	
Nihashi et al. (83)	Probable AD	14 AD	86/97	70/100	No overall differences
Herholz et al. (81)	Probable AD and normal controls	26 AD, 6 NC	–	–	Correlation coefficient of 0.9 between both modalities in temporoparietal and posterior cingulate cortices
Döbert et al. (82)	AD, FTD, VD, Mix and normal controls	9 AD, 1 FTD, 1 VD, 7 mix and 6 NC	91.7/88.9	64.0/84.2	
Ito et al. (84)	Probable AD, MCI due to AD, LBD, FTD	28 AD, 12 MCI, 10 DLB, 5 FTD	77.5–82.5/13.3–40	82.5–87.5/20–33.3	Nearly identical diagnostic performance
Ferreira et al. (86)	Mild AD and normal controls	20 AD, 18 NC	(68–71%)	(68–74%)	
Nadebaum et al. (87)	MCI and dementia	126 patients in total	75.8%/74.3%	42.9%/82.9%	

## Cost-Effectiveness Considerations for SPECT and PET in Dementia

The study of cost-effectiveness for the introduction of functional imaging to dementia diagnostic algorithms have showed contradictory results, mainly due to the scarcity of effective treatments to date. McMahon et al. (88, 89) argue against the inclusion of functional imaging, based on the estimation of quality-adjusted life years and the limited efficacy of cholinesterase inhibitors. According to the authors, any diagnostic test, no matter how perfect, would be incapable of reaching adequate cost-effectiveness thresholds using this methodology unless it has a very low cost. Other authors such as Silverman et al. argue in favor of including functional imaging considering that PET can introduce an increase in diagnostic accuracy of 15% with respect to clinical evaluation, resulting in savings per patient that exceed the cost of a PET study in the United States (90). In their study, the authors consider other costs caused by the disease, such as care expenses, which far exceed those of drug therapy. However, it is important to notice that the exclusion of AD in a patient with dementia does not necessarily imply a reduction in the costs of hospitalization and nursing care, which is fundamentally determined by the functional situation of the patient beyond the etiological diagnosis, since the vast majority of causes of dementia are irreversible. Moulin-Romsee et al. endorsed Silverman's results for the European population. Using the same arguments, they postulate that the diagnostic performance of SPECT in comparison with neuropathological confirmation results in a possible reduction in false diagnoses with respect to conventional algorithms, which is also estimated at 15%, with eventual savings for the health system far superior to the use of PET due to its significantly lower cost (91).

Beyond the discussion of the cost-effectiveness of including functional imaging modalities in the dementia diagnostic algorithm, it seems evident that an earlier or more precise diagnosis will positively affect an already complex situation for the families and the patients suffering from dementia. A more accurate diagnosis will also have important implications for the dementia programs and care systems.

In this context and considering the aforementioned cost-effectiveness dilemmas, the significantly lower cost of SPECT compared to PET (about five times lower in our region) represents a very relevant advantage in favor of the former, particularly in low- and middle-income countries.

## Amyloid Imaging

Research carried out in the last two decades has made possible to detect beta-amyloid deposits *in vivo* by PET. The first radiopharmaceutical used in patients to reveal amyloid, ^11^C-PIB, give the way to several ^18^F-labeled analogs such as florbetapir, florbetaben, and flutemetamol, with considerable advantages in terms of cost and availability due to the longer half-life of ^18^F (12, 92). These ^18^F-labeled radiopharmaceuticals have shown a very good correlation with ^11^C-PIB with high correspondence in visual interpretation (93). High correspondence between ^11^C-PIB uptake and beta-amyloid deposits in neuropathology has been demonstrated in various studies, and there are also similar reports for ^18^F radiopharmaceuticals (93, 94).

However, the investigation on the clinical impact of PET with amyloid tracers is still ongoing. A systematic review by Fantoni et al. found that amyloid PET contributed to diagnostic revision in almost a third of cases and demonstrated value in increasing diagnostic confidence and refining management plans (95). Although it is clear that the technique has an important potential impact in clinical management, it is also recognized that a positive amyloid PET alone is not equivalent to a clinical diagnosis of AD. The presence of amyloid pathology in the brain is insufficient by itself to define the cause of cognitive impairment and must be considered in conjunction with other clinical, laboratory, and imaging elements. The existence of comorbidities such as vascular pathology or depression can still have an important influence on the cognitive deterioration observed in an amyloid PET positive patient. Although current evidence suggests that the majority of individuals with MCI who are amyloid PET positive will progress to AD, the proportion is still not defined, and it is not possible to define when this will happen. However, a negative amyloid PET represents a low risk of progression to AD and may provide a more useful clinical information in patients with MCI, particularly in cases where other potential causes of MCI are present. A similar scenario to that of MCI can be presented in the case of patients who meet diagnostic criteria for possible AD, in whom there is an atypical course of deterioration or comorbid conditions capable of confusing the clinical interpretation.

An important limitation of PET with amyloid tracers is the high prevalence of positive studies in asymptomatic older adults, ranging from 10% at 60 to 70 years to 50% between 80 and 90 years (96). This substantially complicates the establishment of a relationship of causality between amyloid deposits and the presence of deterioration in elderly patients. On the other hand, other pathologies such as amyloid angiopathy and LBD can also present positive studies and cannot be differentiated from AD by amyloid imaging alone. Additionally, around 15% of cases of mild to moderate dementia with a phenotype compatible with AD have no or sparse amyloid deposits (97). Likewise, the technique is not useful in the differential diagnosis of numerous causes of dementia that do not have amyloid deposits, such as the different variants of FTD or LBD, in which approximately one third of cases do not present deposits.

Finally, the performance of amyloid PET in comparison with other lower-cost biomarkers has not been explored enough yet, so it is still difficult to define the most appropriate and cost-effective use strategy within the framework of consensus diagnostic algorithms. The association of amyloid PET with tau and neurodegeneration biomarkers is proposed as the most accurate diagnostic alternative, but the high cost of this approach still limits its applicability in clinical practice.

## SPECT and PET in Clinical and Research Guidelines for the Diagnosis of Dementia

Since the publication of their first guidelines on brain SPECT in the 1990's, the Society of Nuclear Medicine and Molecular Imaging (SNMMI), the American College of Radiologists (ACR) and the European Association of Nuclear Medicine (EANM) recommended the clinical use of the technique in the diagnosis of dementia (98–100). The recommendations of the neurological societies of the United States and Europe at the beginning of the 2000's also considered SPECT, although not in the routine clinical evaluation, but in specific clinical situations in which there were diagnostic doubts and the technique was expected to provide significant additional information. The American Academy of Neurology in 1996 considered SPECT as a useful imaging modality to support the clinical diagnosis of AD based on level IIB evidence (101). As a general concept, the use of perfusion SPECT is recommended in patients with dementia or cognitive impairment of at least 6 months of evolution in which the etiology remains uncertain after a complete clinical evaluation by an experienced physician (including neurological examination, laboratory studies, CT or MRI, and neuropsychological evaluation), when symptoms do not improve within a reasonably short follow-up period after the initial evaluation, and it is reasonable to expect that the information provided by the technique will help clarify the diagnosis or guide future treatment. Patients with advanced stage dementia are excluded. These circumstances can occur in the clinical situations listed in Table 2. FDG PET can be useful in the same situations as perfusion SPECT.

**Table 2:** Clinical situations in which perfusion SPECT or FDG PET can be useful.

Clinical diagnosis of possible AD according to NIA-AA criteria due to atypical clinical presentation, atypical clinical course or coexistence of possible causes.
Clinical diagnosis of persistent or progressive MCI with no clear etiology, especially in the face of coexistence of possible causes.
Early onset progressive dementia (before 65 years of age).
Clinical diagnosis of possible of LBD according to the criteria of the Dementia with Lewy Bodies Consortium.
Differential diagnosis of dementia: to distinguish AD from FTD, LBD, or PD dementia, diagnosis of atypical parkinsonisms, to rule out the association of AD and vascular dementia and to differentiate degenerative dementias from psychiatric pathology.
Persistent cognitive impairment or dementia after brain trauma when MRI does not explain the symptoms.
Low incidence causes of dementia: autoimmune systemic diseases with neuropsychiatric involvement (SLE, Behçet), immune-mediated limbic or extralimbic encephalitis, exposure to neurotoxins, Huntington's disease, Creutzfeld-Jacob disease, HIV encephalopathy, etc.

The approval of the use of FDG PET in dementia by Medicare in 2004 (https://www.cms.gov/medicare-coverage-database), after several negative resolutions, for the differential diagnosis between AD and FTD, has led to the expansion of the use of PET in comparison with SPECT in the United States. This trend is also widespread in high-income countries in Europe. Nowadays, PET is predominantly mentioned in the guidelines, leaving aside SPECT in spite of the important technological advances introduced in the last two decades. The criteria for the diagnosis of AD from NIA-AA consider PET imaging while SPECT is scarcely mentioned (102). The more recent NIA-AA Research Framework for AD emphasizes the importance of biomarkers for the characterization of patients using the A-T-(N) criteria (13), considering PET, MRI, and CSF biomarkers as key players for the study of patients with dementia. Nevertheless, brain SPECT is not mentioned as one of the biomarkers for neurodegeneration, not even as an alternative to PET when it is not available. On the other hand, the recommendations of the NIA-AA for the diagnosis of MCI due to AD recognize the role of SPECT as a biomarker of neurodegeneration (103).

**Figure 3:**
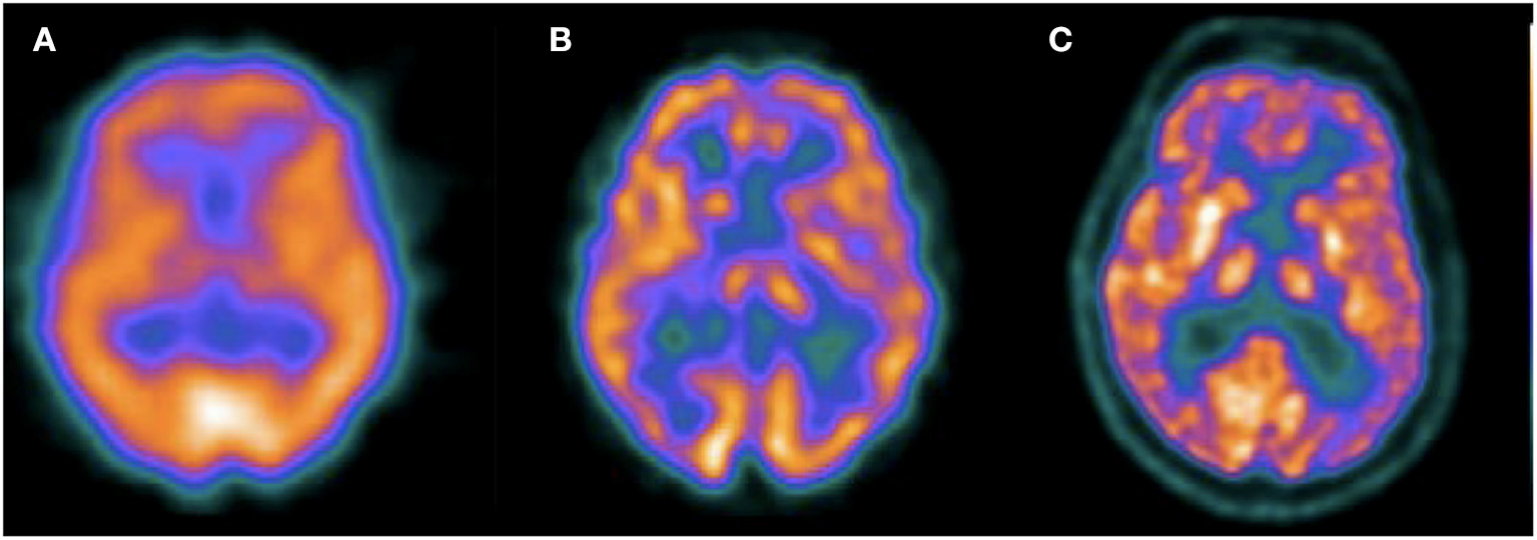
Transaxial slices of perfusion SPECT and ^18^F-FDG PET of different patients. The first image **(A)** corresponds to a perfusion SPECT acquired in a single head gamma camera in 1998 (Sopha DSX rectangular). The second image **(B)** corresponds to a perfusion SPECT with the same radiotracer (^99m^Tc-ECD) acquired in 2009 in a two headed gamma camera, without scatter correction (Mediso Nucline SPIRIT DH-V). The third image **(C)** corresponds to a ^18^F-FDG PET acquired in 2015 (GE Discovery STE). This figure illustrates the significant advances of SPECT image quality in recent years and the comparison with typical ^18^F-FDG images.

It could be argued that SPECT is not part of the current state of the art in the diagnosis of patients with dementia, given the superior quality of PET images. However, the technique has undergone important technical advances since most of the publications that evaluated its usefulness more than 20 years ago. This is exemplified in Figure 3, were typical SPECT images currently available are closer to PET images than old SPECT images obtained in single-head gamma cameras. The review of the diagnostic value of both techniques presented here suggests a much smaller difference in diagnostic performance than that commonly mentioned in the literature, that should be explored in new prospective studies including intra-subject comparison of SPECT and PET using the framework for research in AD as well as clinical follow-up confirmation.

The strategy of performing perfusion/metabolic imaging before amyloid or tau PET can save costs providing a wider spectrum of differential diagnosis of dementia based on the recognition of specific disease patterns and reserving the more complex molecular imaging tools for undetermined cases (Figures 1, 2 show examples of this approach). Even with the advent of the new molecular imaging probes, the role of the biomarkers of neurodegeneration is more valid than ever, since abnormal deposits of proteins like amyloid and tau in the brain can be asymptomatic for many years and do not represent *per se* the presence of dementia. Neurodegeneration represents the necessary condition that signs the beginning of the clinical disease.

In low- and middle-income countries, brain perfusion SPECT is a valid alternative capable to further reduce the costs of the new diagnostic algorithms. High-cost nuclear biomarkers are more difficult to access in these countries, particularly outside the large cities and in users of public health systems (104, 105). This represents not only a limitation for the correct clinical diagnosis but also for the study of populations that are usually underrepresented in scientific research in the field of dementia. SPECT has much higher availability compared to PET (105) and technetium generators needed for the production of SPECT radiotracers are easily transportable to centers in several countries at much lower cost compared to cyclotron-dependent radiotracers. The search for lower-cost biomarkers has also been one of the priorities highlighted by the Latin American and Caribbean Consortium on Dementia (LAC-CD) in a recent publication (106).

## Conclusions

Although amyloid and tau biomarkers have gained ground in recent years and are the current focus of research, neurodegeneration biomarkers continue to play a key role in the diagnosis of dementia. Despite the trend to use PET instead of SPECT in high-income countries, the differences in diagnostic performance between both techniques are subtle, particularly in patients in the clinical stage of dementia, and SPECT has the advantage of wider availability and significantly lower cost. We conclude that SPECT should still be considered an important tool in clinical practice and research in dementia in low- and middle-income countries.

## Author Contributions

RF conceived the presented idea. RF and AD wrote the manuscript and contributed to the final version.

## Conflict of Interest

The authors declare that the research was conducted in the absence of any commercial or financial relationships that could be construed as a potential conflict of interest.
